# Brief environmental enrichment elicits metaplasticity of hippocampal synaptic potentiation *in vivo*

**DOI:** 10.3389/fnbeh.2012.00085

**Published:** 2012-12-14

**Authors:** Arne Buschler, Denise Manahan-Vaughan

**Affiliations:** ^1^Department of Neurophysiology, Faculty of Medicine, Ruhr University BochumBochum, Germany; ^2^International Graduate School for Neuroscience, Ruhr University BochumBochum, Germany

**Keywords:** *in vivo*, mouse, hippocampus, CA1, LTD, LTP, synaptic plasticity, environmental enrichment

## Abstract

Long-term environmental enrichment (EE) elicits enduring effects on the adult brain, including altered synaptic plasticity. Synaptic plasticity may underlie memory formation and includes robust (>24 h) and weak (<2 h) forms of long-term potentiation (LTP) and long-term depression (LTD). Most studies of the effect of EE on synaptic efficacy have examined the consequences of very prolonged EE-exposure. It is unclear whether brief exposure to EE can alter synaptic plasticity. Clarifying this issue could help develop strategies to address cognitive deficits arising from neglect in children or adults. We assessed whether short-term EE elicits alterations in hippocampal synaptic plasticity and if social context may play a role. Adult mice were exposed to EE for 14 consecutive days. We found that robust late-LTP (>24 h) and short-term depression (<2 h) at Schaffer-collateral-CA1 synapses in freely behaving mice were unaltered, whereas early-LTP (E-LTP, <2 h) was significantly enhanced by EE. Effects were transient: E-LTP returned to control levels 1 week after cessation of EE. Six weeks later, animals were re-exposed to EE for 14 days. Under these conditions, E-LTP was facilitated into L-LTP (>24 h), suggesting that metaplasticity was induced during the first EE experience and that EE-mediated modifications are cumulative. Effects were absent in mice that underwent solitary enrichment or were group-housed without EE. These data suggest that EE in naïve animals strengthens E-LTP, and also promotes L-LTP in animals that underwent EE in the past. This indicates that brief exposure to EE, particularly under social conditions can elicit lasting positive effects on synaptic strength that may have beneficial consequences for cognition that depends on synaptic plasticity.

## Introduction

In rodents, environmental enrichment (EE) mimics the circumstances of a stimulating and interesting living environment that is conducive to learning and cognition. During EE, toys, nesting material, tubes, huts, and running wheels provide sensory, cognitive and motor stimulation for rodents that are normally not part of standard animal housing (Rampon and Tsien, 2000; Van Praag et al., 2000; Nithianantharajah and Hannan, 2006). Throughout the whole brain, structural (Turner et al., 2003; Leggio et al., 2005; Bose et al., 2010; Rasin et al., 2011) and molecular (Rampon et al., 2000a; Nithianantharajah et al., 2004; Angelucci et al., 2009; Mainardi et al., 2010) changes occur after EE that are accompanied by increased brain weight (Collins, 1970). Particularly the hippocampus shows a variety of alterations after EE. These are mirrored in improvement of performance of hippocampus-dependent tasks, such as the water maze (Kempermann et al., 1997), contextual fear conditioning (Tang et al., 2001), and radial arm maze (Huang et al., 2006). However, EE does not only affect hippocampus-dependent learning behavior. In addition, hippocampal neurons show increased complexity and spine density after EE (Rampon et al., 2000b; Bindu et al., 2007; Fréchette et al., 2009; Beauquis et al., 2010). Neurogenesis and survival of newborn neurons in the dentate gyrus (DG) are enhanced after exposure to EE that includes a running wheel (Kempermann et al., 1997; Bruel-Jungerman et al., 2005; Segovia et al., 2006). On the molecular level, the expression of plasticity-related factors is altered after EE. These include postsynaptic density protein 95 (PSD95), synaptophysin (Nithianantharajah et al., 2004), Ca^2+^/calmodulin-dependent kinase II (CaMKII), cyclic adenosine monophosphate response element-binding protein (CREB) (Huang et al., 2006), the GluA1 subunit of alpha-amino-3-hydroxy-5-methyl-4-isoxazolepropionic acid-receptor (AMPAR), and the N-methyl-D-aspartate-receptor (NMDAR) subunits GluN2A and GluN2B (Tang et al., 2001). Moreover, bidirectional synaptic plasticity in the hippocampus, which relates to learning and memory (Morris, 1989; Bear and Malenka, 1994; Braunewell and Manahan-Vaughan, 2001; Kemp and Manahan-Vaughan, 2007), is altered after EE. *In vivo* studies show that synaptic plasticity, including long-term potentiation (LTP) (Gruart et al., 2006) and long-term depression (LTD) (Goh and Manahan-Vaughan, 2012) in the murine CA1-area, is closely related to learning. Numerous *in vitro* studies show strengthened CA1-LTP (Duffy et al., 2001; Artola et al., 2006; Huang et al., 2006) and -LTD (Artola et al., 2006) in rodents after EE.

All of the abovementioned studies on EE used paradigms that exposed the animals to EE for prolonged periods. These important studies laid the groundwork for our understanding of the role of adequate sensory and cognitive stimuli for improved behavioral learning, hippocampal structure, and synaptic plasticity, but none of these studies examined this phenomenon *in vivo*. Our goal was to examine if much briefer periods of EE can elicit positive effects on hippocampal synaptic plasticity, and if effects can be sustained. Focus was made on synaptic plasticity in the freely behaving mouse. Clarification of these aspects is of importance given current discussions as to effective strategies to address cognitive deprivation and/or the effects of neglect on children and elderly adults. A recent study indicated that activities such as dancing in a social environment can have marked effects on cognitive abilities in elderly humans (Kattenstroth et al., 2010). This also provokes the question as to the necessity and importance of social contact in such strategies. In this study we therefore investigated the impact of a relatively short period of 14 days of consecutive EE on synaptic plasticity in the hippocampal CA1 region. We then explored whether re-exposure to EE after a period of 6 weeks had similar or different effects on plasticity. Furthermore, we assessed the role of social environment in these effects. Finally, we investigated the influence of repeated EE, and dissociated inanimate from social stimulation. We found that short-term EE transiently strengthens weak synaptic potentiation *in vivo*, but re-exposure to EE facilitates the expression of robust LTP that lasts for over 24 h. Effects are only evident in animals that undergo EE in a social environment. These data suggest that brief EE under social conditions can have potently positive effects on synaptic plasticity.

## Methods

### Animals

The present study was carried out in accordance with the European Communities Council Directive of September 22nd, 2010 (2010/63/EU) for care of laboratory animals and after approval of the local ethics committee (Bezirksamt Arnsberg, Germany). All efforts were made to minimize animal suffering and to reduce the number of animals. Male C57/BL6 mice (Charles River, Germany) were used in all of the experiments. All mice attained the minimum weight of 22 g before being subjected to surgical electrode implantation. The mice were housed individually in a temperature- and humidity-controlled vivarium with a constant 12-h light-dark cycle (lights on from 6 a.m. to 6 p.m.) where they had access to food and water *ad libitum*. All surgical procedures and experiments were conducted during the day.

### Surgery and implantation of electrodes

The implantation of chronic electrodes into the Schaffer collateral (SC)-CA1 pathway of adult mice was carried out as described before (Buschler et al., 2012; Goh and Manahan-Vaughan, 2012). Briefly, at the age of 7–8 weeks, mice were anaesthetized (sodium pentobarbital, 60 mg/kg) and underwent stereotactic surgery. According to coordinates obtained from a mouse brain atlas (Paxinos and Watson, 2007) a bipolar stimulation electrode was placed in the SC pathway of the right dorsal hippocampus [anterior–posterior (AP): −2.0 mm, mediolateral (ML): 2.0 mm, and dorsoventral (DV): ca. −1.4 mm from the brain surface] and a monopolar recording electrode was placed into the ipsilateral CA1 *stratum radiatum* (AP: −1.9, ML: 1.4, and DV: ca. −1.2 mm from the brain surface). Electrodes (polyurethane-coated stainless steel wire, 100 μm diameter; Gündel, BioMedical Instruments, Germany) were inserted through a single trephine hole (ca. 1.4 mm). Ground and reference electrodes (stainless steel, A-M Systems, USA) were attached to contralateral anchor screws that were fixed in two additional holes. During the surgery, test-pulse stimulation was used to adjust the depth of stimulation and recording electrodes. After the appropriate response was identified, all electrodes were assembled to a 6-pin socket (Conrad Electronic SE, Germany) and fixed with dental acrylic (J. Morita Europe GmbH, Germany; Heraeus Kulzer GmbH, Germany). Mice were allowed to recover for 7–10 days before experiments were conducted. During the post-surgical period animals were monitored for infection and received analgesic treatment.

### Measurement of evoked potentials

Twenty-four hours before an electrophysiological experiment, mice were placed into the recording chamber [20 (L) × 20 (W) × 30 (H) cm] to ensure familiarization to the environment. During the recordings, mice had full access to food and water. A flexible cable connected to the animals' socket and a swivel connector allowed unhindered movement of the mice. Field excitatory postsynaptic potentials (fEPSPs) in the CA1 region were evoked by stimulating the SC pathway using biphasic square pulses of 0.2 ms duration per half-wave, generated by a constant stimulus isolation unit (World Precision Instruments, USA). Evoked responses were recorded at a test-pulse frequency of 0.025 Hz, amplified via an AC-amplifier (A-M Systems, USA) and digitized through an AD converter (Cambridge Electronic Design, UK). Five consecutive test-pulse responses, evoked at 40 s intervals, were averaged and the slope of the initial negative deflection was taken as representative of synaptic transmission. During a given experiment, all values were referenced to the mean of the first 6 averaged responses that were recorded in 5 min intervals (baseline = 30 min) and expressed in percentage (± standard error of the mean). Typically, high-frequency stimulation (HFS) or low-frequency stimulation (LFS) was applied after the baseline. Five minutes after the plasticity-induction protocol, test-pulse stimulation was commenced and continued for 4 h (at 5 min intervals for 15 min, thereafter at 15 min intervals). On the following day, responses were recorded roughly 24 h after commencement of the experiment and were continued for 1 h at 15 min intervals. Prior to each experiment the maximal evoked response was determined during an input–output (IO) curve determination (maximal intensity 125 μA). For test-pulse stimulation or HFS, the intensity eliciting 40% maximal response was used. IO-properties were also used to evaluate changes in basal synaptic transmission after environmental manipulation. Early-LTP (E-LTP) was defined a potention that lasted for no longer than 2 h, whereas late-LTP was defined as potentiation that persisted for over 24 h. Short-term depression was defined as synaptic depression that lasted for less than 2 h.

To induce synaptic potentiation, patterned 100 Hz stimulation consisting of a single train of 50 pulses, or 2 × 50 pulses [5 min inter-train interval (ITI)], was used. A single 50 pulse sequence typically elicits weak potentiation, whereas 2 × 50 pulses induce robust L-LTP (Buschler et al., 2012). LFS of 900 pulses at 3 Hz was applied at 70% of the maximal response intensity. Throughout the experiments animals were resting. During patterned stimulation all mice were awake and had open eyes, signifying alertness. Experiments and stimulation were also always conducted at the same time of day. By this means we could exclude that general changes in behavioral state, related to the sleep-wake cycle, could alter plasticity or general evoked responses (Bramham and Srebro, 1989; Leung et al., 2003). All animals were first tested in a “baseline” experiment without any patterned simulation to ensure that the recordings were stable (data not shown).

Throughout the experiments the electroencephalogram (EEG) of each animal was monitored continuously for seizure activity. At the end of the study, the mouse brains were removed and histological verification of electrode localization was carried out (Buschler et al., 2012). Frozen brain sections (30 μm) were stained according to the Nissl method using 1% toluidine blue (Bock, 1989), and then examined using a light microscope. Individuals in which incorrect electrode localizations or hippocampal malformations were found were excluded from the data analysis.

### Housing conditions

Under standard conditions (cage size: 267 × 207 × 140 mm, floor area: 370 cm^2^) mice were reared solitarily after the surgery. During EE, mice were continuously reared in groups of 5–6 age-matched animals, or were housed solitarily, in an enrichment cage (cage size: 595 × 380 × 200 mm, floor area: 1820 cm^2^) for 14 consecutive days. During EE all groups/individuals were exposed to the same number of objects. Three pieces of tunnel, one house, nesting material, hanging climbing-structure of four plastic-mesh-balls, two movable balls, climbing rings, and a running wheel were added to the cage. All objects, except the running wheel were rearranged every 3rd day. Re-exposure to EE took place 6 weeks after cessation of initial EE. The 2nd EE-period also was conducted for 14 consecutive days using similar EE-objects. Social rearing in the absence of additional environmental stimuli was carried out by group housing (cage size: 425 × 266 × 185 mm, floor area: 800 cm^2^) of six age-matched mice. Synaptic plasticity was tested before the respective rearing paradigm and directly after cessation. To analyze the longevity of changes, synaptic plasticity was re-assessed 1 week after cessation of EE.

The following cohorts were used during this study: four groups of mice were used to assess the influence of group-EE on synaptic plasticity. A cohort of six mice underwent HFS to induce robust L-LTP (100 Hz, 2 × 50 pulses), and two additional cohorts of five mice underwent HFS to induce E-LTP (100 Hz, 50 pulses). One of these E-LTP-cohorts (five mice) was re-exposed to EE. Another group of six mice was tested for STD, using LFS (3 Hz, 300 pulses). Six individually housed mice experienced solitary-EE and an additional cohort of six mice was reared socially for 2 weeks.

### Statistical analysis

Analysis of variance (ANOVA) with repeated measures followed by *post-hoc* Fisher LSD test and student's *t*-test was used to evaluate the changes of synaptic strength between the groups. The analysis was applied on the data points after patterned stimulation until the end of the experiment. In some cases the first 3 data points after HFS were additionally analyzed. The significance level was set to *p* < 0.05.

## Results

### Synaptic potentiation following environmental enrichment in groups

Our first question was whether a brief period of EE can modify synaptic plasticity, given reports that long period of EE are effective. For this purpose, mice underwent continuous EE for 14 consecutive days.

**Figure 1:**
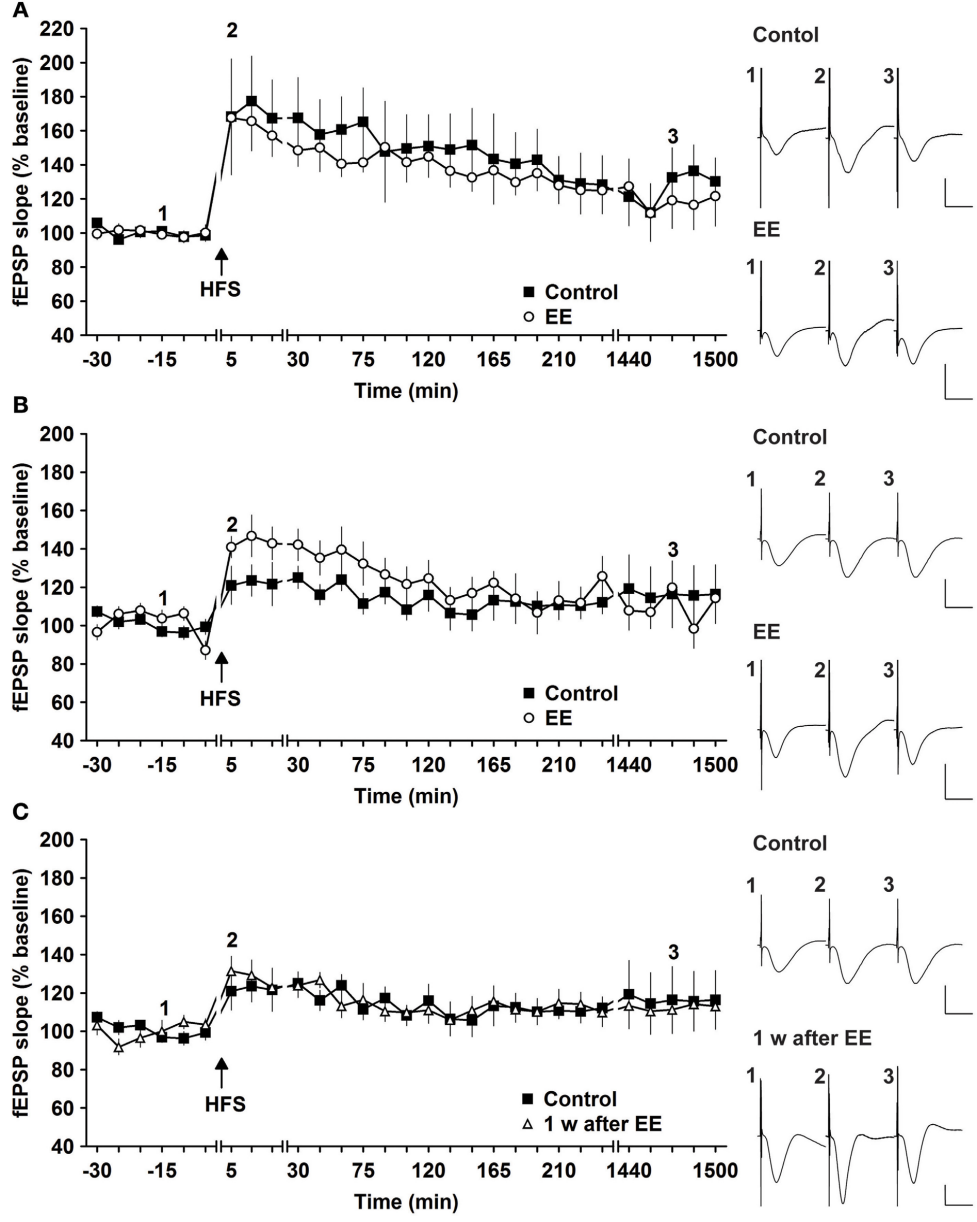
**Robust and weak potentiation are differently affected by environmental enrichment. (A)** Strong high frequency stimulation (HFS, 2 trains of 50 pulses) induces robust late-long-term potentiation (L-LTP) that lasts for over 24 h in controls. Environmental enrichment (EE) for 14 days does not alter the profile of this LTP. **(B)** The magnitude of early-long-term potentiation (E-LTP), that is evoked by weak high frequency stimulation (HFS, 50 pulses at 100 Hz) is significantly increased following 14 days of environmental enrichment (EE) under group-housing conditions. **(C)** Seven days after cessation of EE, fEPSP values returned to control (pre-EE) levels. Time-point of high frequency stimulation (HFS) is marked with a filled arrow. Insets: Analog examples of field potentials (averages of five consecutive sweeps) from typical experiments at the time indicated by the numbers. Horizontal bar: 10 ms, vertical bar: 2 mV.

The same animals were tested for magnitude and longevity of synaptic potentiation prior to commencement of EE. Here, HFS of 2 × 50 pulses at 100 Hz induced robust L-LTP that lasted for over 24 h, as reported previously (Buschler et al., 2012). Strikingly, 14 days of EE did not alter the profile of L-LTP [Figure 1A, *n* = 6, ANOVA: *F*_(1, 10)_ = 0.28, *p* > 0.6 compared to controls, *n* = 6].

One possibility was that the L-LTP evoked was already so robust that little improvement could be achieved. In fact, previous results indicate that HFS using the abovementioned protocol evokes saturated LTP at the murine SC-CA1 pathway *in vivo*, since increasing the stimulation strength does not enable increases in LTP (Buschler et al., 2012). For this reason we explored whether the same EE paradigm would affect weaker potentiation.

HFS of 1 × 50 pulses at 100 Hz elicited E-LTP that lasted for ca. 2 h in animals prior to EE exposure (Figure 1B). Interestingly, EE for 14 days significantly increased the magnitude of potentiation during the initial 120 min after HFS [Figures 1B, 3, *n* = 10, ANOVA: *F*_(1, 18)_ = 5.49, *p* > 0.031]. No change in the duration of LTP occurred, however, and 1 week after cessation of EE, application of HFS resulted in E-LTP that was equivalent to controls [Figure 1C, *n* = 10, ANOVA: *F*_(1, 18)_ = 0.68, *p* > 0.42].

**Figure 2:**
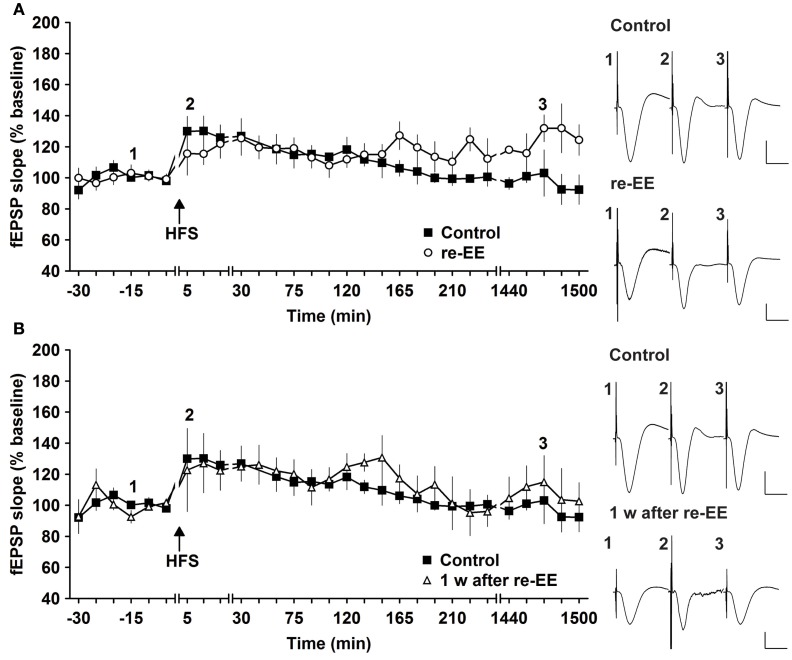
**Early-long-term potentiation is facilitated into late-long-term potentiation by re-exposure to environmental enrichment.** Six weeks after cessation of environmental enrichment (EE), animals were once more exposed to EE for 14 days. **(A)** In this case, E-LTP was prolonged into L-LTP that lasted for over 24 h. **(B)** Seven days after cessation of EE, fEPSP values returned to control (pre-EE) levels. Time-point of high frequency stimulation (HFS) is marked with a filled arrow. Insets: Analog examples of field potentials (averages of five consecutive sweeps) from typical experiments at the time indicated by the numbers. Horizontal bar: 10 ms, vertical bar: 2 mV.

**Figure 3:**
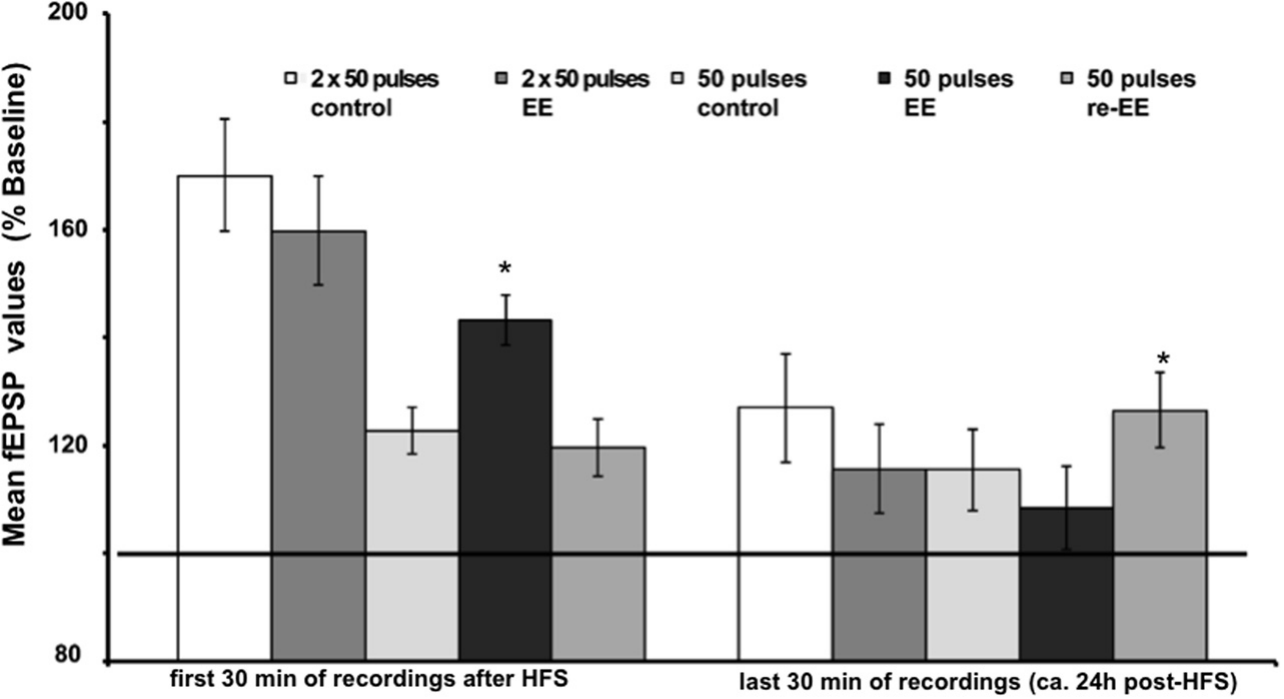
**Mean fEPSP values of initial 30 min and last 30 min of recordings of potentiation after EE.** Bar charts show the mean fEPSP values recorded either in the first 30 min immediately after application of HFS, or the last 30 min (ca. 24 h after HFS) in mice stimulated with either one application of 50 pulses at 100 Hz, or two applications of 50 pulses at 100 Hz. LTP elicited with the stronger protocol (2 × 50 pulses) lasts for over 24 h but does not respond to EE (compared to non-enriched controls). LTP elicited with the weaker protocol (1 × 50 pulses) is no longer present 24 h after HFS in controls. However, the initial 14 day exposure to EE (1st EE) enhances the magnitude of the first 30 min of E-LTP. Re-exposure to EE (2nd EE) 6 weeks after 1st EE results in a significant prolongation of late (L-)LTP in EE-treated mice compared to controls that is evident 24 h after HFS. The reference baseline is represented by a black bar (100%). Asterisk denotes significant effect.

To assess whether repeated exposure to EE has different effects on LTP, one group of animals was re-exposed to 2 weeks of EE exactly 6 weeks after conclusion of the first 14 day EE exposure (*n* = 5). Under these conditions, E-LTP was prolonged into L-LTP(>24 h) [Figures 2A, 3, *n* = 5, ANOVA: *F*_(1, 8)_ = 7.72, *p* < 0.024]. Effects were not persistent: 1 week after once more returning to standard housing, HFS elicited E-LTP that was equivalent to controls [Figure 2B, *n* = 5, ANOVA: *F*_(1, 8)_ = 1.22, *p* > 0.3]. These observations suggest that re-exposure to EE (following a 6 week interim of no EE) elicits more potent effects on synaptic potentiation compared to naïve exposure to EE.

### Synaptic depression following environmental enrichment

**Figure 4:**
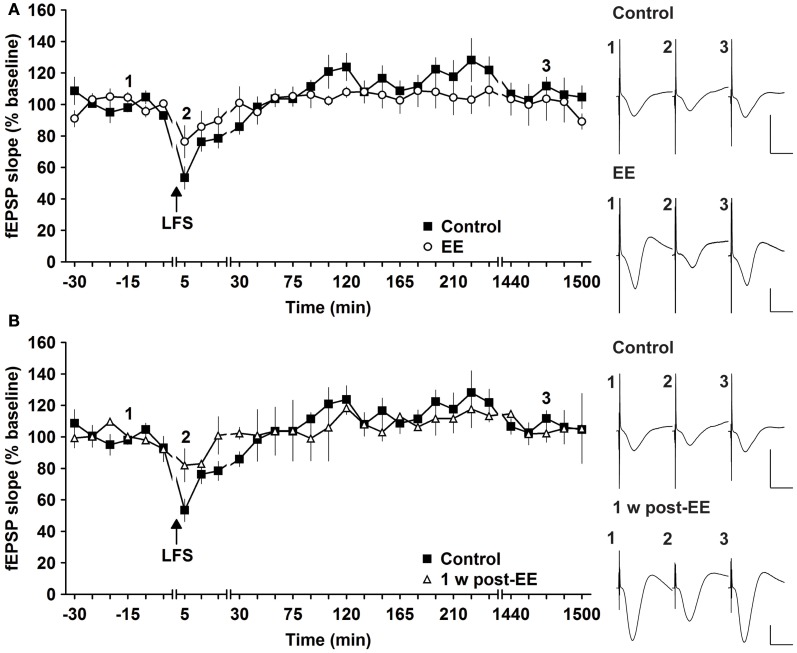
**Environmental enrichment does not affect short-term depression. (A)** Short-term depression (STD) induced by 3 Hz (900 pulses) is not affected by 14 days of group environmental enrichment (EE). **(B)** One week after cessation of EE, no alterations were seen **(B)**. Effects were compared with STD evoked in the same animals before EE was commenced. Time-point of low frequency stimulation (LFS) is marked with a filled arrow. Insets: Analog examples of field potentials (averages of five consecutive sweeps) from typical experiments at the time indicated by the numbers. Horizontal bar: 10 ms, vertical bar: 2 mV.

Most studies that address effects of EE in mice have examined LTP (Duffy et al., 2001; Huang et al., 2006; Li et al., 2006; Arai et al., 2009). To clarify if synaptic depression is also affected by EE we used LFS of 900 pulses at 3 Hz, which has been shown to reliably induce STD in freely behaving mice (Buschler et al., 2012). LFS given prior to commencement of EE for 14 days, elicited STD (<2 h) (Figure 4A). EE for 14 days had no effect on the profile of STD obtained [Figure 4A, *n* = 5, ANOVA: *F*_(1, 8)_ = 0.22, *p* > 0.64]. Similarly, 1 week after cessation of EE responses were equivalent to controls [Figure 4B, ANOVA: *F*_(1, 8)_ = 0.16, *p* > 0.69]. These results indicate that STD is not altered after short-term EE.

### Solitary enrichment and social housing

**Figure 5:**
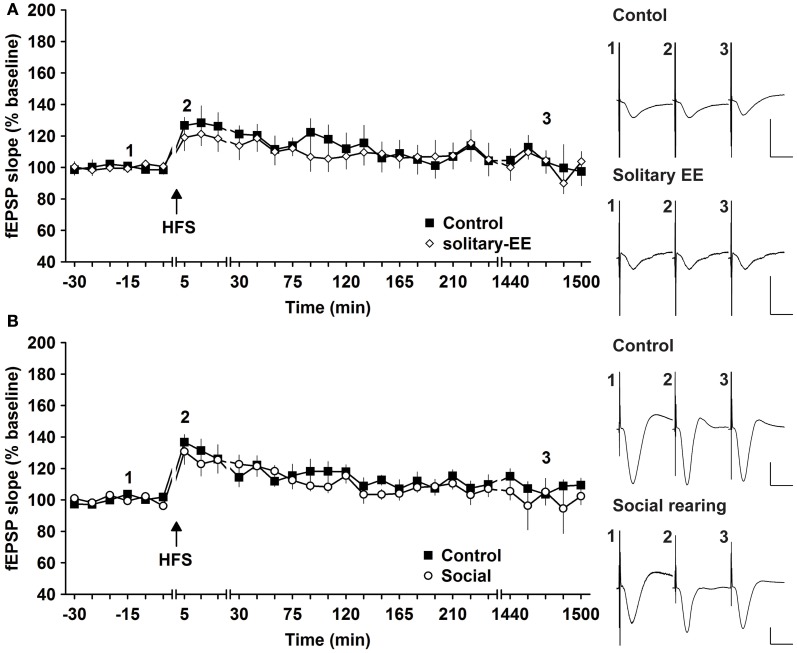
**Environmental enrichment under solitary conditions, or social housing in absence of enrichment, has no impact on synaptic potentiation. (A)** Assessment of E-LTP after housing under enriched conditions without social interaction revealed no alteration compared to control E-LTP before environmental enrichment (EE). **(B)** Furthermore, group housing in the absence of EE had no effect on E-LTP. Time-point of high frequency stimulation (HFS) is marked with a filled arrow. Insets: Analog examples of field potentials (averages of five consecutive sweeps) from typical experiments at the time indicated by the numbers. Horizontal bar: 10 ms, vertical bar: 2 mV.

Social factors can strongly influence the responsiveness of human subjects to enriching behavior (Kattenstroth et al., 2010). We therefore explored whether it was the social housing or the EE itself that drove the synaptic changes we observed. We compared HFS-induced synaptic potentiation in animals that were exposed to EE in solitary conditions or in groups. HFS given to animals that underwent solitary EE elicited STP that was not different to controls [Figure 5A, *n* = 6, ANOVA: *F*_(1, 10)_ = 0.16, *p* > 0.73]. Thus, group EE (Figure 1A) and not solitary EE (Figure 5A) led to facilitation of E-LTP into L-LTP. We next assessed whether group housing alone could also facilitate synaptic plasticity by examining the influence of prolonged group housing in the absence of EE. Compared to controls, 2 weeks of group housing did not alter STP [Figure 5B, *n* = 6, ANOVA: *F*_(1, 10)_ = 0.33, *p* > 0.57]. These results indicate that it is a combination of social contact and EE that facilitates synaptic plasticity.

### Basal synaptic transmission following enrichment and social housing

**Figure 6:**
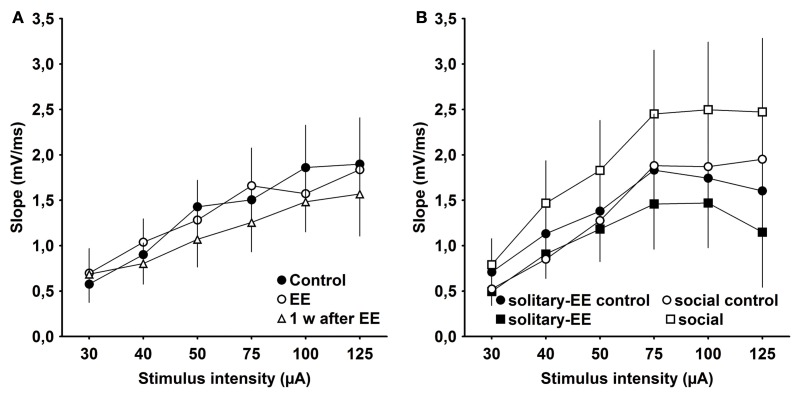
**Input–output analysis of evoked responses reveals no significant changes in excitability as a result of environmental enrichment. (A)** Input/output (IO) properties remained stable between control, environmental enrichment (EE) and 1 week (w) post-EE. **(B)** After solitary-EE and social rearing without EE, IO curves were not significantly different compared to controls.

One possibility is that the effects of EE that we observed are related to changes in synaptic excitability rather than to plasticity *per se*. To examine this, we assessed IO properties in animals that were housed under standard conditions (control), experienced EE in group housing, EE in solitary conditions, social rearing without EE, or in animals tested 1 week after conclusion of EE. We observed that IO properties remained stable between control animals, EE animals (group conditions) and animals 1 week post-EE (Figure 6A, Control × EE: *p* > 0.90, Control × 1 week after EE: *p* > 0.39, EE × 1 week after EE: *p* > 0.46). Furthermore, IO properties were equivalent after solitary-EE and social rearing without EE (Figure 6B, solitary-control × solitary-EE: *p* > 0.35, social-control × social: *p* > 0.09). This supports the conclusion that changes in excitability do not underlie the facilitation of LTP observed following EE.

## Discussion

This study demonstrates that brief EE potently strengthens hippocampal synaptic plasticity. Effects are prolonged: whereas first exposure to EE for 14 days enhances the magnitude of E-LTP, re-exposure to EE 6 weeks after conclusion of the first spell results in a significant facilitation of E-LTP into L-LTP that lasts for well over 24 h. In contrast, synaptic depression at the CA3-CA1 synapse was not affected significantly by 2 weeks of EE. In contrast, rats that were enriched for 5 weeks exhibited significantly stronger LTD after EE (Artola et al., 2006). Prolonged access to EE conditions in the Artola study may explain the different results. However, an important point is that in general, mice are more resistant than rats to expressing LTD following afferent stimulation to the hippocampus (Manahan-Vaughan, 2000; Buschler et al., 2012; Goh and Manahan-Vaughan, 2012). Thus, the lack of effect of EE on LTD may relate to the general behavioral and physiological conditions needed to elicit synaptic depression in this rodent species.

The effects of EE on LTP were only evident if EE occurred under social conditions: EE experienced in solitary housing did not facilitate LTP. These findings are in line with the early enrichment experiments of Rosenzweig et al. (1978) that revealed that social stimulation alone was insufficient to explain the alteration in cortical RNA-expression and acetylcholinesterase activity that occurred after 30 days of EE in rats. In contrast, the water-maze performance of solitarily-enriched and group-enriched rats was enhanced to a similar extent after 9 weeks of exposure to a running wheel (Schrijver et al., 2002). This may rather reflect the specific effect of exercise on neurogenesis and synaptic viability, however, (Van Praag et al., 1999a,b; Stranahan et al., 2006; Kobilo et al., 2011).

Both social recognition memory (Kogan et al., 2000) and L-LTP (Barco et al., 2002; Nguyen and Woo, 2003) require the activation of CREB in murine CA1. Hippocampus-dependent social recognition memory (of rodent identity and conspecifics) is sustained for periods of up to 7 days in mice, but is disrupted by long periods of isolation (Kogan et al., 2000). This finding offers interesting parallels to our own and may reflect the social nature of rodents. Our data suggest that EE alone is not sufficient to facilitate LTP, rather the enrichment must occur in social groups of animals. These findings have interesting implications, and offer functional insights into observations in studies with humans that show that cognitive benefits of enriched living conditions occur if conducted under social conditions (Kattenstroth et al., 2010).

Since IO-properties did not shift after EE, the facilitation of LTP we observed is unlikely to occur due to altered basal synaptic transmission and/or excitability. Rather, effects appear to be directly related to a modulation of LTP. Facilitation of synaptic strength due to learning-related experience in rodents has been previously reported (Manahan-Vaughan and Braunewell, 1999; Kemp and Manahan-Vaughan, 2004) and a delayed rise of fEPSP responses has been described in mice after reconsolidation of object-recognition memory (Clarke et al., 2010), suggesting that learning experience affects hippocampal field responses. Indeed, novel spatial learning experience potently facilitates synaptic plasticity in mice *in vivo* (Goh and Manahan-Vaughan, 2012).

The stimulation paradigm we used induces N-methyl-D-aspartate receptor (NMDAR)-dependent potentiation (Buschler et al., 2012) and NMDARs are crucial for hippocampus-dependent learning and synaptic plasticity (Davis et al., 1992). Strikingly, robust L-LTP was not affected by EE, however. This is not surprising given the fact that it is likely that this form of LTP was already close to saturation levels (Buschler et al., 2012). LTP may reflect information storage that underlies specific forms of hippocampus-dependent learning (Kemp and Manahan-Vaughan, 2007). The observation that E-LTP can be reinforced by brief EE, and that a repeated exposure to EE later results in persistent LTP (L-LTP) provokes the tantalizing possibility that EE can reinforce cognition that underlies memory. This is the first study that suggests that brief EE can have such beneficial effects. Other studies, where EE was conducted for longer periods than used here, also reported enhanced LTP (Duffy et al., 2001; Artola et al., 2006; Huang et al., 2006). A question which remains unanswered is what could underlie this long-term effect of brief EE on a mechanistic level. One possibility is that metaplastic changes are elicited by EE that endure long after the initial effects on E-LTP can be detected. Metaplasticity (Deisseroth et al., 1995; Abraham and Bear, 1996; Tsien et al., 1996) can strongly influence the direction of change in synaptic strength (Kemp and Manahan-Vaughan, 2005; Zhang et al., 2005). It comprises mechanistic changes that alter the ability of a synapse to respond to a subsequent attempt to induce synaptic plasticity. Factors such as priming of NMDARs (Mockett et al., 2002), of the metabotropic glutamate receptors (Manahan-Vaughan et al., 1996) or the behavioral state of the animal (Manahan-Vaughan and Braunewell, 1999) all can underlie metaplasticity. Thus, the brief periods of EE may have triggered metaplastic changes in the synapse. Effects appear to be cumulative: the initial EE may have initiated changes in the mechanistic profile of LTP that were added to by the second EE exposure. Nonetheless, the question remains as to what could bring about such potent changes.

EE offers stimulating and interesting living conditions that are likely to activate neuromodulatory systems such as the dopaminergic system that responds to reward, or the noradrenergic system that is stimulated by novelty. Both the dopaminergic (Kulla and Manahan-Vaughan, 2000; Lemon and Manahan-Vaughan, 2006, 2011; Lemon et al., 2009) and the noradrenergic system (Kemp and Manahan-Vaughan, 2008; Hagena and Manahan-Vaughan, 2012) exert a potent neuromodulatory regulation of synaptic plasticity, that is also evident during learning conditions. On the molecular level, one possible substrate for EE is the transcription factor *egr-1/zif268*. Mice, suffering from *egr-1* deficiency display impairment of L-LTP, memory consolidation and reconsolidation (Bozon et al., 2002). On the other hand, *egr-1/zif268* becomes upregulated after EE in rats (Koh et al., 2005). Transcription of plasticity-related proteins has also been reported after EE (Nithianantharajah et al., 2004) including key proteins such as CREB (Huang et al., 2006) and glutamate receptor subunits (Tang et al., 2001). These changes, if prolonged, could support enhanced LTP.

Another consideration is that because the second exposure to EE occurred 6 weeks after the conclusion of the first exposure, the more potent effects of the 2nd exposure may have more to do with the increased age of the mice than to do with the re-exposure to EE This seems unlikely, given the relatively young age of the mice, but it is worth mentioning that long-term EE elicits an improvement of both synaptic plasticity and learning performance in both adult and aged rodents (Huang et al., 2006; Bouet et al., 2011; Freret et al., 2012).

Behavioral state can potently affect the outcome of an attempt to induce synaptic plasticity (Bramham and Srebro, 1989; Leung et al., 2003; Tsanov and Manahan-Vaughan, 2007a,b). This can relate to motion (Leung et al., 2003), the sleep-wake cycle (Bramham and Srebro, 1989; Tsanov and Manahan-Vaughan, 2007a), or circadian phase (Tsanov and Manahan-Vaughan, 2007b). For this reason, we went to lengths to ensure that our animals were in equivalent behavioral states during attempts to induce plasticity: experiments were always commenced at the same time of day, plasticity stimulation was always given before noon, the animals were awake, motionless and resting, but had open eyes during plasticity-inducing stimulation. By this means we can assume that the role of behavioral state under our comparative conditions was negligible. One cannot exclude, however, that solitary mice are less active than group-reared mice, and that effects due to differences in exercise levels (Titterness et al., 2011) may have played a role in the outcome of EE under social and non-social conditions.

In conclusion, our data support that short-term EE has significant effects on synaptic potentiation, but a repeat of EE several weeks later has potent effects on the longevity of LTP. Given the relationship between LTP and hippocampus-dependent learning (Kemp and Manahan-Vaughan, 2004, 2007; Whitlock et al., 2006) this suggests that EE given once or repeatedly may have beneficial effects on hippocampus-dependent cognition. Effects are only apparent if EE is conducted under social conditions. To wit: “use it, or lose it, but don't do it alone.”

### Conflict of interest statement

The authors declare that the research was conducted in the absence of any commercial or financial relationships that could be construed as a potential conflict of interest.
